# Clinical and Laboratory Presentation of Hydatid Disease: A Study From Northeast India

**DOI:** 10.7759/cureus.10260

**Published:** 2020-09-05

**Authors:** Arup Baruah, Kalyan Sarma, Bhupen Barman, Pranjal Phukan, Chandan Nath, Polina Boruah, Purnima Rajkhowa, Mriganka Baruah, Anirban Dutta, Narang Naku

**Affiliations:** 1 Department of General Surgery, North Eastern Indira Gandhi Regional Institute of Health and Medical Sciences, Shillong, IND; 2 Department of Radiology, North Eastern Indira Gandhi Regional Institute of Health and Medical Sciences, Shillong, IND; 3 Department of Medicine, North Eastern Indira Gandhi Regional Institute of Health and Medical Sciences, Shillong, IND; 4 Department of Biochemistry, North Eastern Indira Gandhi Regional Institute of Health and Medical Sciences, Shillong, IND; 5 Department of Microbiology, Silchar Medical College, Silchar, IND; 6 Department of Biochemistry, Employees State Insurance Corporation Medical College, Kolkata, IND

**Keywords:** hydatid cyst, echinococcosis, hydatid disease, pair (percutaneous aspiration, echinococcus

## Abstract

Introduction

Hydatid disease is an immense health problem in developing countries. The diagnosis of hydatid cyst is often difficult because of its protean manifestations. Our objective was to evaluate the various clinical and laboratory presentations of hydatid disease and various modalities of treatment from a tertiary care center.

Materials and methods

We reviewed the clinical and laboratory features of patients presenting with hydatid cyst through retrospective analysis from January 2018 to December 2019 from a tertiary care hospital in northeast India.

Results

Of the 26 adult patients with hydatid cysts who were part of the study, 14 (53.8%) were males and 12 (46.2%) were females. The mean age was 34.6 years. The most common site of involvement was the liver (69%) followed by lung (19.2%) and brain (7.7%). Palpable mass in the right upper quadrant of the abdomen was the most common symptom (88.3%) for liver hydatid cyst followed by pain abdomen (66.6%). Systemic symptoms like fever and weakness were present in most of the patients. The majority of patients (80%) were from rural areas.

Conclusion

Hydatid cysts present with varied symptomatology. History of exposure to infected animals may not be present. A high degree of clinical suspicion combined with meticulous history and clinical examination supported by laboratory investigations are required for its diagnosis.

## Introduction

Hydatid disease is an infectious parasitic disease caused by the larval stage of the *Echinococcus granulosus* complex, *E. multilocularis* or* E. vogeli*. These parasites are found worldwide with high prevalence in the Middle East, the Mediterranean region, central Asia, China, eastern Africa, and parts of South America with increasing prevalence in the recent past [1]. There are sporadic reports of cases from different regions of India. However, the majority of cases have been recorded from southern and western India viz. Andhra Pradesh, Saurashtra, and Tamil Nadu with a prevalence of around 10-15% [2-6]. The liver is the most common site for hydatid disease (75% of cases), followed by lungs (15%), and occasionally being reported in the kidney, spleen, peritoneal cavity, and the skin and muscles (2-3% each); and rarely in the heart, brain, vertebral column, and ovaries (1% or less each) [7]. Diagnosis of hydatid disease is based on the epidemiological background of patients, clinical signs and symptoms, and radiographic and related imaging studies. Serodiagnostic assays can be particularly helpful and detection of antibody to specific echinococcal antigens has the highest degree of specificity. Treatment of hydatid cyst is based on considerations of the location, size, clinical signs and symptoms, and individual characteristics of the patient. Surgery has traditionally been the gold standard of treatment. Percutaneous aspiration, infusion of scolicidal agents, and reaspiration (PAIR) can be used in special circumstances. Medical therapy with albendazole can be used as primary therapy as well as prophylaxis therapy before PAIR or surgical procedure. With this background, the present study was conducted to analyze the presenting symptomatology according to the involvement of different sites and/or organ systems and to analyze the treatment modalities given to patients with hydatid disease.

## Materials and methods

The study was designed as a retrospective single-center, observational cohort study, wherein 26 patients (aged 18 or more) with hydatid cyst were retrospectively included from the departments of surgery, general medicine, and radiology & imaging of North Eastern Indira Gandhi Regional Institute of Health & Medical Sciences (NEIGRIHMS), Shillong, in northeast India. The diagnosis was confirmed by ultrasonography (USG) abdomen, computed axial tomography (CT), and magnetic resonance imaging (MRI) scan of affected organs and by morphological and microscopic examination of the operative specimen. Immunodiagnosis for the detection of circulating *E. granulosus* antigens in serum by enzyme linked immunosorbent assay (ELISA) was done in all patients. The age, sex, history of contact with dog, clinical presentation, treatment advised, operative and postoperative management (both surgery and PAIR) of patients were recorded and analyzed. Data were coded and summarized using Statistical Product and Service Solutions (SPSS) Statistics for Windows, Version 21.0 (IBM Corp., Armonk, NY). Quantitative variables were described using mean ± standard deviation and categorical data by using frequency and percentage.

## Results

A total of 26 patients were diagnosed with hydatid cyst in our hospital between January 2018 and December 2019. Of these, 14 (53.8%) were males and 12 (46.2%) were females (male:female ratio = 1.2:1). The patients’ ages ranged from 19 to 67 years (mean age = 34.6 years). Most of the patients (35.7%) were in the age group of 31 to 40 years. The majority of patients were from rural areas (80%) and the remaining (20%) were from urban areas. History of contact with dogs was available in nine (34.6%) cases.

The most common organ involved was liver (69.2%), followed by lung (19.2%), brain (7.7%), and spinal cord (3.8%). Multiple organ involvement was found in five (19.2%) cases. The involvement of spleen was found in four (15.38%) patients. In three of these cases, there was presence of a hydatid cyst of the liver and in one case with a spinal cyst. The most common symptomatic presentation among patients with a liver hydatid cyst was a palpable mass in the right upper quadrant (83.3%). Although a high index of suspicion is required to diagnose hydatid cyst on time, yet, a constellation of presentations such as abdominal pain (66.6%), loss of appetite (55.5%), weight loss (44.4%), and jaundice (33.3%) was found to be useful clinical indicators of liver hydatid cyst (Table 1).

**Table 1:** Descriptive features of the study population SD: standard deviation, CNS: central nervous system

Parameters	Variables	Number (percentage)
Age (years) (Mean±SD)	34.6±12.4	
Sex	Male	14 (53.8%)
	Female	12 (46.2%)
Residence	Rural	20 (80%)
	Urban	06 (20%)
Site of involvement (N=26)	Liver	18 (69.2%)
	Lung	05 (19.2%)
	CNS	02 (7.7%)
	Spine	01 (3.8%)
Abdominal symptoms (N=18)	Lump in abdomen	15 (83.3%)
	Pain abdomen	12 (66.6%)
	Loss of appetite	10 (55.5%)
	Weight loss	8 (44.4%)
	Jaundice	6 (33.3%)
Chest symptoms (N=5)	Cough	4 (80%)
	Chest pain	4 (80%)
	Hemoptysis	1 (20%)
Miscellaneous (N=26)	Fever	18 (69.2%)
	Weakness	15 (57.7%)

A complete hemogram with differential count was performed in all patients. Anemia was reported (defined as <12 gm/dl in female and <13 gm/dl in male) in 12 (46.1%) patients. Eosinophilia, defined as more than 500 eosinophils per cubic millimeter was present in 11 (42.3%) patients. The liver function test was carried out in all 26 cases. Elevated bilirubin level (>2 mg/dl) was noted in eight (20.7%) cases. Serum alkaline phosphatase was elevated in 12 (46.1%) cases (Table 2).

**Table 2:** Hematological and biochemical characteristics of study population SD: standard deviation; Hb: hemoglobin; AEC: absolute eosinophil count; ESR: erythrocyte sedimentation rate; SGOT: serum glutamic-oxaloacetic transaminase; SGPT: serum glutamic pyruvic transaminase; ALP: alkaline phosphatase

Parameters	Mean ± SD
Hb, g/dl	12.08 ± 2.18
AEC	521.36 ± 269.70
ESR, mm at the end of 1^st^ hour	43.44 ± 20.72
Bilirubin, mg/dl	1.42 ± 0.82
SGOT, IU/L	29.24 ± 9.18
SGPT, IU/L	23.08 ± 7.01
ALP, IU/L	175.84 ± 93.63

**Figure 1:**
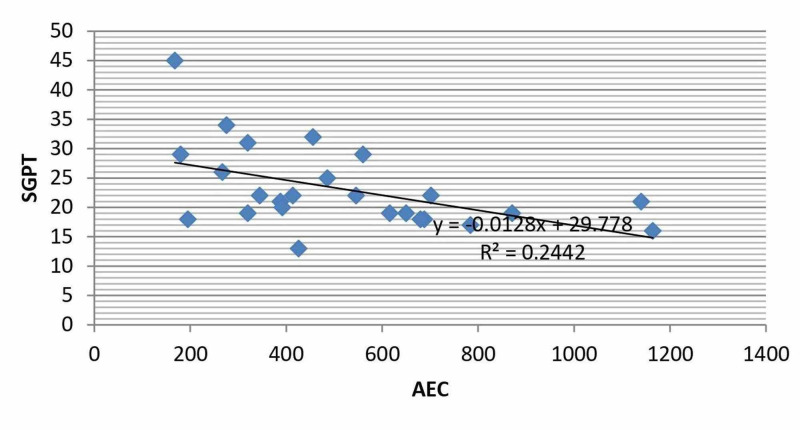
Correlation between AEC and Serum SGPT in patients with Hydatid Cyst AEC: absolute eosinophil count; SGPT: serum glutamic pyruvic transaminase

The correlation coefficients (r) of absolute eosinophil count (AEC) with various laboratory parameters showed a significant (p<0.05) negative correlation between AEC and serum glutamic pyruvic transaminase (SGPT) level (Figure 1). 

No correlation of AEC with other laboratory parameters like hemoglobin (Hb), erythrocyte sedimentation rate (ESR), total bilirubin, platelet count and serum glutamic-oxaloacetic transaminase (SGOT) level was noted. As evident from Table 3, correlation coefficient (r) value with respect to these parameters is less than the critical value of 0.423 (at the significance of p=0.05), with 20 degrees of freedom.

**Table 3:** Correlation coefficient of AEC with various laboratory parameters AEC: absolute eosinophil count; Hb: hemoglobin; ESR: erythrocyte sedimentation rate; SGOT: serum glutamic-oxaloacetic transaminase; SGPT: serum glutamic pyruvic transaminase; NS: Non-significant

Parameter	The correlation coefficient (r)	Significance at P=0.05 (Critical value of r is 0.423 with 20 degrees of freedom)
AEC Vs Hb	-0.00406	NS
AEC Vs ESR	0.157036	NS
AEC Vs Platelet Count	-0.24261	NS
AEC Vs Bilirubin	-0.00028	NS
AEC Vs SGOT	-0.38199	NS
AEC Vs SGPT	-0.49418	Significant

USG of the abdomen was done in all patients. The liver hydatid cysts were diagnosed with USG in all suspected cases showing a sensitivity of 100%. Most liver cysts were found in the right lobe with a predilection for the inferior surface. Multiple liver cysts were present in three (11.54%) cases. The maximum number of hepatic cysts observed in a patient was three. In five cases, the liver was concomitantly involved with other viscera like spleen (n=3) and pelvis (n=2).

In the present study, 12 (46.15%) cases of liver hydatid disease underwent surgery. Three cases underwent additional splenectomy for concurrent hydatid cysts in the spleen. Open cyst evacuation and drainage of the cyst was done in all the patients who underwent surgery for liver hydatid cyst. As a precautionary measure, the entire field of operation was covered with hypertonic (20%) saline-soaked mops before opening the cavity of the cyst to guard against the spillage of the cyst content. The cyst cavity was then opened carefully, its contents aspirated completely with a suction device and 20% hypertonic saline solution introduced into its cavity to deactivate the protoscoleces and germinative membrane. After carefully clearing all of its contents, the cyst cavity was inspected for any possible bile leak. No such cases were found in the present study. After confirming the absence of biliary communication, the residual cyst cavity was obliterated by omentoplasty in nine cases and capitonnage along with drains in three cases. Two patients with the drainage procedure had wound sepsis requiring prolonged hospital stay before successful discharge.

PAIR was performed in four cases of liver hydatid cyst. All the patients received oral albendazole 400 mg twice daily for four weeks to minimize the dissemination during the procedure. Hydrocortisone 100 mg was injected to all the patients on the day of the procedure. Under local anaesthesia with all aseptic and antiseptic procedure, a small skin incision was made by using a scalpel blade (number 11). To puncture the cyst a 16G liver biopsy needle was used. The puncture needle was then exchanged with a KMP catheter (Cook Medical, Bloomington, IN, USA) over the 0.035'' Terumo guidewire (Terumo Medical Corporation, Somerset, NJ, USA). Approximately 80% of the cyst content was aspirated and the cyst cavity was filled with an equal amount of 50% nonionic contrast. A cystogram was done in two planes to check for any communications between the cyst cavity and nearby structures like the biliary tract. The content of the cyst cavity was aspirated again and filled with absolute alcohol (98% ethyl alcohol) in a volume equaling two-thirds of the aspirate. The alcohol was aspirated out from the cyst cavity. All patients were kept for continuous monitoring for at least 24 hours to avoid early complication. They were later followed-up at first month and sixth month and the cysts were evaluated by USG examination. The success of the PAIR was measured with the following parameters: (i) reduction in size and volume of the cyst (ii) reduction in irregularity, and thickening of the cyst wall (iii) disappearance of the fluid component and (iv) solidification of the cyst.

The lung was the second most commonly involved (19.2%) organ after the liver. Chest pain, chronic cough with expectoration, dyspnea, hemoptysis, and fever were common presenting symptoms noted in patients with pulmonary hydatid cyst. Routine chest X-ray was done in all patients with pulmonary symptoms. Pulmonary hydatid cyst was seen as rounded homogenous and spherical shadows in a chest film. Contrast-enhanced computed tomography (CECT) of chest was done to confirm the diagnosis in all five cases of pulmonary hydatid cyst. Four out of five patients had cysts in their right lung while one patient had a cyst in their left lung. All five cases had solitary pulmonary cysts. One of the patients with pulmonary hydatid cyst had associated pulmonary tuberculosis. In four of the cases including the case with associated tuberculosis, decortication and marsupialisation of cyst was done. Postoperatively, patients were put on intercostal drainage tubes which were later removed. One of the patients developed pneumothorax postoperatively which later resolved without further complication. All the patients recovered well.

**Figure 2:**
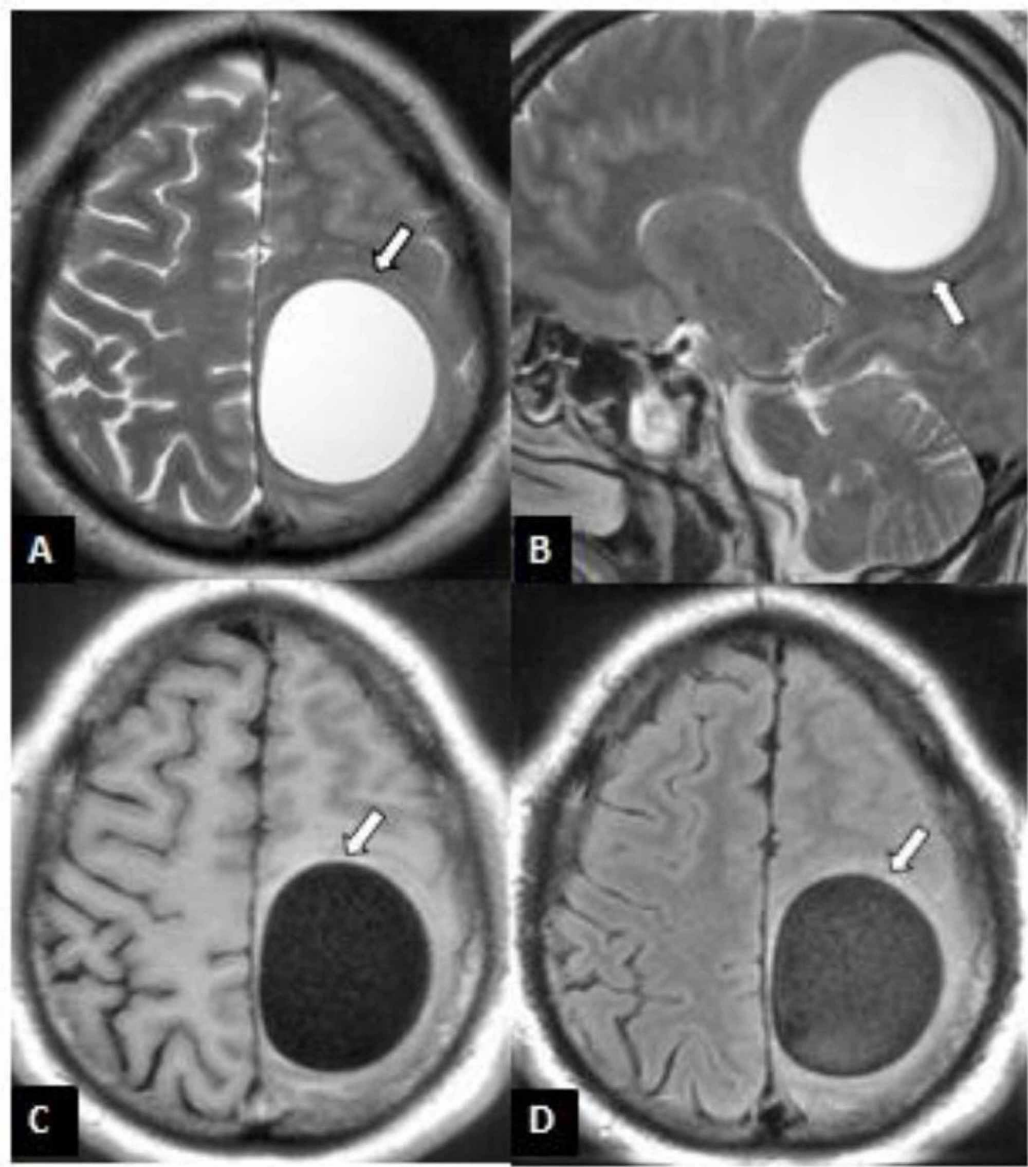
MRI brain of a 28-year-old patient with cerebral hydatid cyst A: Axial T2WI; B: Sagittal T2WI; C:Axial T1WI; D: Axial FLAIR image demonstrating a large uniloculated hydatid cyst (white arrows) in the left fronto-parietal region with minimal overall mass effect relative to size of cyst MRI: magnetic resonance imaging; T2WI: T2 weighted image; T1WI: T1 weighted image; FLAIR: fluid-attenuated inversion recovery

Intracranial hydatid disease is rare and was reported in two cases of our cohort. Fronto-parietal and parieto-occipital regions of the brain were involved in the two patients respectively. Both patients had presented with progressive symptoms consisting of headaches, diminished vision, confusion and focal neurological deficits. Brain MRI revealed two intra-axial multilocular cystic lesions in the fronto-parietal and parieto-occipital regions (Figure 2). The two patients underwent operations and the lesions were removed without rupture. Medical therapy with albendazole was started. Neurological symptoms disappeared a few weeks after the surgeries. 

**Figure 3:**
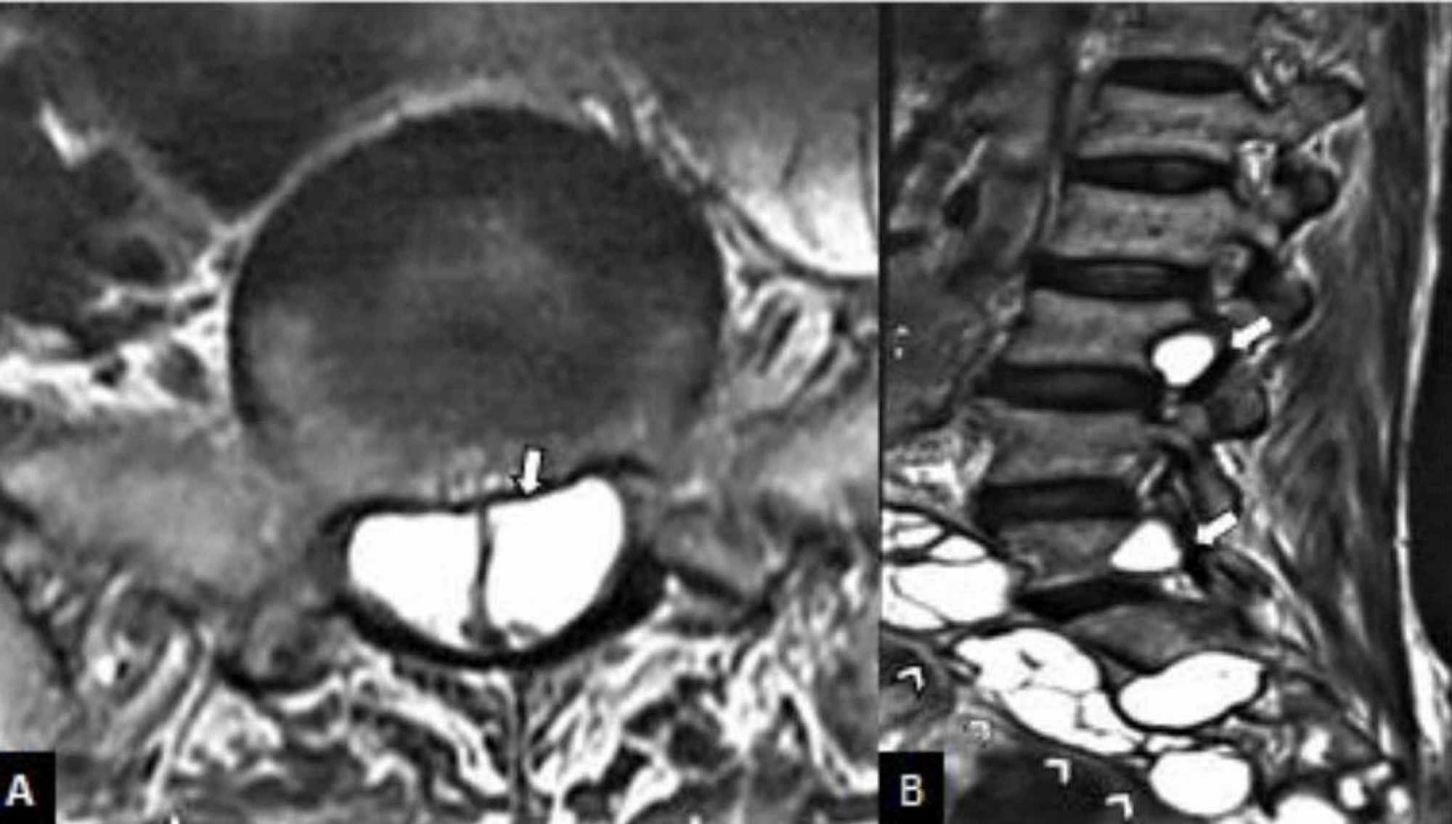
MRI of lumbosacral spine of 36-year-old patient with spinal hydatid cyst A: axial T2WI; B: sagittal T2WI MRI of lumbosacral spine showing multiple intradural (white arrows) and paraspinal hydatid cysts (white arrow heads) MRI: magnetic resonance imagine; T2WI: T2 weighted image

One patient had a hydatid cyst in the spinal cord along with multiple cysts in other visceral organs like liver and spleen (Figure 3). The patient presented with compressive myelopathy and was discharged on oral albendazole due to refusal of surgical treatment. 

## Discussion

Echinococcosis (hydatid disease) is a zoonosis caused by the larval stages of tapeworms of the genus *Echinococcus* that form pathogenic cysts in humans. Animal hosts of the parasites comprise wild carnivores, farm and domestic animals, and other small mammals including rodents. Infection of humans occurs by ingestion of *Echinococcus* eggs that are most commonly shed in faeces of dogs and several wild canids, such as foxes, wolves, jackals, and coyotes.

Hydatid disease has been recognized as a global public health problem. It has been found in all sheep-raising countries including India. The highest prevalence is reported mostly from the southern part of India viz. Andhra Pradesh and Tamil Nadu [2-6]. On reviewing literature from the rest of the world, hydatid disease has been strongly linked with the sheep-raising industry and dogs which act as intermediate and definite hosts. In the present study, none of the patients were involved with sheep rearing as an occupation. However, most of the patients were from a rural background, where exposure to domestic animals like cattle including sheep and stray dogs is quite common. History of direct contact with dogs was present in nine (34.61%) cases; it may be safely postulated that those patients may have acquired the disease either by transitory handling of dogs or by consuming raw vegetables and water contaminated with excreta of an infected dog; a similar view has also been expressed by previous studies [2-6].

Hydatid cysts can occur at any age group but are commonly prevalent in young people at the peak of their productive lives. This is reflected in this study as the majority of our patients were in the third and fourth decades of life, which is consistent with other studies [7-9]. Some studies like Palanivelu et al. (5:1) have reported higher male preponderance [10]. However in our study, the gender distribution was almost equal with slight female preponderance which is similar to previous studies [11-13]. In the present study, eosinophillia was found in 42% of cases and jaundice was found in 20.7% of cases. Christians et al. reported approximately one-third of patients had eosinophilia and 20% had jaundice and high bilirubin [14].

In our study liver was found most commonly involved in both sexes. This is in congruence with observations of other studies [15-16]. A few studies have reported equal or even higher involvement of lung [17-18]. In the present study, the distribution of liver hydatid was right lobe 16 (84.2%), left lobe one (5.2%), and both lobes two cases (10.5%). The reason for higher involvement of the right side may be on account of greater blood supply to the right lobe than the left lobe of the liver as described by Maingot in 2004 in about 80% of his cohorts of hydatid cyst of the liver [14]. Hepatic cysts are slow-growing and usually only manifest after achieving sufficient size. In the present study, hydatid presented as right upper quadrant (RUQ) swelling on local examination and revealed a tense mass in connection with the liver.

Surgery remains the major treatment of choice for liver hydatid cysts. However, there is considerable disagreement regarding the technique of surgery, i.e. complete removal of endocyst or conservative or radical surgery. Demirci et al. in their series of 260 patients with hydatid cysts of the liver reported development of postoperative biliary fistula in 3.5% patients in a group of 87 patients treated with radical procedures as compared with a 27.5% rate in the group of 173 patients treated by external drainage [19].

In our study, abdominal USG proved diagnostic in all cases. In the study conducted by Balik et al. (1999), USG showed a diagnostic accuracy of 97.7% [9]. In our study, the CECT scan of the abdomen was done in 12 (48%) cases. It was 100% diagnostic for liver hydatid cyst. In the study conducted by Balik et al. the diagnostic accuracy of CECT was found to be 100% [9]. MRI can help identify the rim and differentiate this diagnosis from other encapsulated liver lesions. Irregularities of the rim border, which can be accepted as signs of a partial detachment, are more reliably demonstrated with MRI than with CECT or USG of the abdomen [20].

Pulmonary hydatid cyst is the second most common site after liver and mostly (60%) affects in the right lower lobe of the lung, although it may even involve left upper lobe and in few cases, it may be multiple or bilateral [11,21,22]. Pulmonary hydatid cysts often remain asymptomatic for years and incidentally diagnosed with chest X-ray. Subsequently, patients may present with various clinical sign and symptoms like cough with sputum, dyspnea, hemoptysis, chest pain depending on cyst site and size [12]. All five patients of pulmonary hydatid cyst in the current study presented with cough with expectoration, three of them had a fever and one had hemoptysis. One of the patients had co-existent sputum positive pulmonary tuberculosis. Co-infection of tuberculosis and hydatid cyst is an important public health problem and as many as six cases of co-infection with tuberculosis and hydatid cyst have been described [13]. Although details of the pathogenesis of this association are not known, there are postulated hypotheses of low socioeconomic status and unhygienic practice and immunogenic theory. As the chronicity of hydatid cyst increases, the immune profile of the host appears to change from a Th1 to Th2 response. With a suppressed Th1 immune profile, the host’s ability to detect and respond to viruses, bacteria, and other pathogens is impaired [23].

Spinal hydatid disease, the most common form of bone involvement, is usually difficult to distinguish from tuberculous spondylitis, osteomyelitis, and abscess [24]. The typical characteristics of spinal hydatid cyst are lack of osteoporosis and sclerosis of the vertebra, sparing of disk spaces and vertebral bodies, the involvement of contiguous rib with paraspinal extension. Although CT allows precise assessment of structural bony lesions and identifies hydatid cyst calcification, MR imaging is superior in demonstrating the involvement of neural structures [25,26].

Intracranial hydatid cyst, a rare and fatal presentation of Echinococcosis (<1%), occurs by a direct infestation of the brain by larvae [27]. The most common site for intracranial hydatid cyst is supratentorial and parietal lobe, mostly in the distribution of the middle cerebral artery [25].On imaging, hydatid cyst characteristically appears as a well-defined, thin-walled cerebrospinal fluid-signal cyst, which is usually solitary and may contain multiple daughter cysts. There is usually no surrounding edema unless the parenchyma is secondarily infected. The cyst either does not enhance or enhances very thinly and may have peripheral calcification. The important differential diagnoses in imaging studies include neurocysticercosis as well as various brain cysts like arachnoid cyst and epidermoid cyst. The presence of multiple small daughter endocysts usually helps to differentiate hydatid from other cysts. These may not be appreciated on CT scans, so MRI is indicated in patients with cystic lesions.

The simultaneous involvement of more than two organs is observed in around 20% of patients [28]. In the present study, multiple organ involvement was found in five (19.2%) cases. Spleen is the third most common site for hydatid cyst where it may present with a wide variation of findings. Splenic involvement may be primary (involves the spleen only) or secondary (with multiple organ involvement) and may be in the form of solitary or multiple cysts [29,30]. Splenic hydatid cyst was reported in four cases in the present study and all four cases had secondary involvement with solitary splenic cysts.

## Conclusions

To conclude, we catalogue the clinical and laboratory presentation of hydatid cyst cases diagnosed in our center over a period of two years. Hydatid cyst is a public health problem, more so in developing countries. As most cases were from rural areas it seems the local mode of hydatid infection is by eating raw vegetables and drinking water contaminated by infected dogs. A high degree of clinical suspicion is required for its diagnosis and subsequent management. History of the patient and meticulous clinical examination like the presence of cystic swellings anywhere in the body especially at rare and unusual sites can provide valuable pointers towards its diagnosis, especially when corroborated with biochemical and serological findings. The role of imaging modalities like ultrasonography and computed tomography of the abdomen as components of the work-up of such patients has become indispensable. These modalities are not only helpful for diagnosing the usual cases of hydatid cyst but are also helpful for staging and detecting the atypical cases. Early treatment is mandatory to avoid general complications that are directly related to the duration of the cysts.
